# Effect of Process Control Parameters on the Filtration Performance of PAN–CTAB Nanofiber/Nanonet Web Combined with Meltblown Nonwoven

**DOI:** 10.3390/polym13203591

**Published:** 2021-10-19

**Authors:** Hyo Kyoung Kang, Hyun Ju Oh, Jung Yeon Kim, Hak Yong Kim, Yeong Og Choi

**Affiliations:** 1 Advanced Textile R&D Department, Korea Institute of Industrial Technology, Ansan 15588, Korea; hkkang@kitech.re.kr (H.K.K.); hjoh33@kitech.re.kr (H.J.O.); cobalt98@kitech.re.kr (J.Y.K.); 2 Department of Organic Materials and Fiber Engineering, Jeonbuk National University, Jeonju 54896, Korea; 3 Department of Nano Convergence Engineering, Jeonbuk National University, Jeonju 54896, Korea

**Keywords:** nanonet, polyacrylonitrile, surfactant, electrospinning, meltblown, nanofiber/nanonet

## Abstract

Nanofibers have potential applications as filters for particles with diameters <10 μm owing to their large specific surface area, macropores, and controllable geometry or diameter. The filtration efficiency can be increased by creating nanonets (<50 nm) whose diameter is smaller than that of nanofibers. This study investigates the effect of process conditions on the generation of nanonet structures from a polyacrylonitrile (PAN) solution containing cation surfactants; in addition, the filtration performance is analyzed. The applied electrospinning voltage and the electrostatic treatment of meltblown polypropylene (used as a substrate) are the most influential process parameters of nanonet formation. Electrospun polyacrylonitrile–cetylmethylammonium bromide (PAN–CTAB) showed a nanofiber/nanonet structure and improved thermal and mechanical properties compared with those of the electrospun PAN. The pore size distribution and filter efficiency of the PAN nanofiber web and PAN–CTAB nanofiber/nanonet web with meltblown were measured. The resulting PAN–CTAB nanofiber/nanonet air filter showed a high filtration efficiency of 99% and a low pressure drop of 7.7 mmH_2_O at an air flow rate of 80 L/min. The process control methods for the nanonet structures studied herein provide a new approach for developing functional materials for air-filtration applications.

## 1. Introduction

Air pollution threatens human health and the environment worldwide [1]. The manufacturing, transportation, and construction industries are some of the major contributors of air pollution as they release toxic waste and fine particles into the atmosphere [2,3]. High concentrations of particulate matter (PM) in the air are a primary cause of respiratory diseases [4,5,6]. PM, which consists of extremely small particles and liquid droplets [7], is classified into coarse (2.5–10 μm, PM10), fine (0.1–2.5 μm, PM2.5), and ultrafine (<0.1 μm, PM0.1) PM [8]. In particular, PM2.5 can easily penetrate the human lungs and bronchi after a short period of exposure [9,10]. To prevent the inhalation or ingestion of PM2.5, researchers recommend the use of a mask filter for personal protection during outdoor activities; further, a window screen and purification filter should be used for indoor air quality protection.

Fibrous filters are generally commonly used for air filtration because they have a network support structure, can trap PM, and are made of tortuous pore channels for air transport [11]. In general, fibrous filters can be classified into meltblown (MB), spunbond, electrospun, and needle-punch filters [12]. Among them, conventional filters such as nonwoven needle-punch, glass-fiber, and spunbond filters have large fiber diameters and are suitable for use as coarse filters [13]. By contrast, meltblown filters and electrospun filters have small diameters and pores; therefore, they are used for fine particle filtration. MB nonwoven fabrics have a large surface area per unit weight and remarkable barrier properties, with a pore size of 0.5–10 μm and an average diameter of 1–2 μm [14]. To increase the filtration efficiency, the fiber diameter must be decreased; however, there are limitations in the equipment design and process [15].

Electrospinning is a simple and versatile top–down spinning method capable of producing nano-to-submicron nonwoven filters from polymer and polymeric mixture solutions [16,17,18]. Electrospun nonwoven fabrics have a large surface area and porosity, making them significantly more effective in filtering out particles than their conventional counterparts [19,20]. In previous studies, we produced nano-sized nonwoven fabric filters by electrospinning various polymers, namely poly(vinyl alcohol) [21], poly(vinyl alcohol)/lignin [22], polystyrene [23], polyacrylonitrile (PAN) [24], polylmethylmethacrylate (PMMA) [24], polyethersulfone (PES) [25], polyurethane (PU) [26], and poly(lactic acid) [27]. The use of electrospinning for the fabrication of nano-sized nonwoven fabric filters improved the filtration performance over that of micrometer-sized nonwoven filters. Researchers have been conducting studies to further improve the efficiency of nanofiber filters [1,28]. To further reduce the diameter of electrospun nanofibers, electrospinning/net generation technology is being studied by controlling the process parameters. For instance, Shichao et al. [28] produced a PMIA nanofiber/net air filter with a filtration efficiency of 99.9% at a pressure drop of 92 Pa via electrospinning by mixing a surfactant with poly(m-phenylene isophthalamide). In addition, polyurethane (PU) was electrospun into a 20-nm-diameter nonwoven piece of fabric with a wide range of Steiner tree structures. An air filter with filtering efficiencies of 99% and 99.73% for PM1–0.5 and PM2.5–1, respectively, at a pressure drop of 28 Pa was fabricated [2]. The effect of collector type on net formation was also studied [29]. Hsiao et al. [30] added a surfactant to poly(vinylidene fluoride) to fabricate a nanofiber/nanonet and revealed that the filtration efficiency of the nonwoven fabric was 99.985% at 32 L/min and the pressure drop was 66.7 Pa. Furthermore, the effect of applied voltage on the formation of nanonets was studied. Several studies were conducted on air filters with nonwoven fabrics made of nanofibers/nanonets, and they achieved high efficiencies and low pressure drops.

PAN is a polar polymer that has a high dipole moment of 3.6 D; therefore, it can trap particulate pollutants in the air relatively easily [19,31]. In addition to a high filtration efficiency, it exhibits good ultraviolet resistance, weatherability, and chemical resistance [19]. PAN electrospun nanofiber membranes with these advantages are good materials for air filter media. As a related study, Lakshmanan et al. [32] produced an air filter media laminated with an electrospun web by adding a surfactant to the PAN solution. However, a nanonet structure was not observed. In fact, there are few studies related to the formation of nanonet structures in PAN polymers. Thus, there is substantial scope for manufacturing functional filter media by taking advantage of the characteristics of PAN and nanonets.

**Figure 1:**
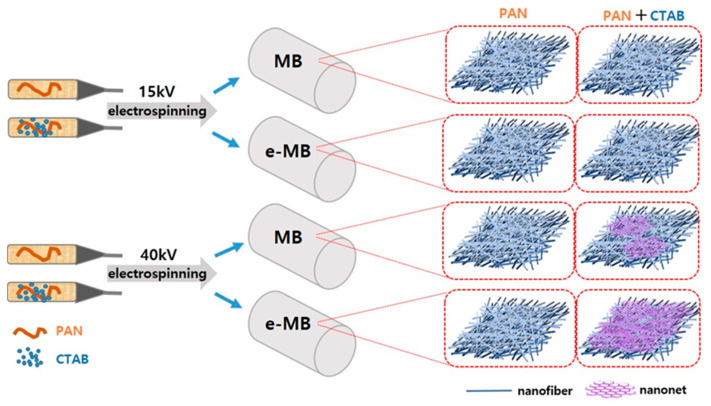
Schematic of the fabrication of PAN–CTAB nanofiber/nanonet web on electrostatically treated meltblown (e-MB) and discharged MB nonwoven filter.

In this study, we investigated the formation conditions of nanonet structures in PAN nanofibers to obtain optimal filter media. The nanofiber–nanonet structure was achieved by controlling electrospinning process parameters such as the cationic additive, applied voltage, and electrostatically treated MB (e-MB) substrate (Figure 1). The morphology, thermal properties, and mechanical properties of the PAN nanofibers and PAN-CTAB nanofiber/nanonet web were analyzed. The pore size distribution and filter efficiency of PAN-CTAB/MB and pristine PAN/MB filter media were measured by varying the nanofiber basis weights. After fabricating a face mask using the PAN-CTAB nanofiber/nanonet web, the filtration efficiency and pressure drop were characterized under an increasing flow rate.

## 2. Materials and Methods

### 2.1. Materials

Analytical-grade polyacrylonitrile (PAN, average *M*w = 150,000 g mol^−1^) was purchased from Sigma-Aldrich. Cetylmethylammonium bromide (CTAB), *N*,*N*-dimethyl formamide (DMF), and isopropyl alcohol (IPA) were obtained from Dae-Jung Chemical & Metals Co., Ltd., Siheung, Korea. The e-MB material used as a substrate was provided by Cheong-Jung clean Co., Ltd., Jincheon, Korea. All chemicals were used without further treatment.

### 2.2. Preparation of Filter Media

The PAN solution was prepared by dissolving 1.739 g of PAN in 20 mL of DMF and stirring for 10 h at 26 °C. The PAN–CTAB solution was prepared by adding 1.3 wt% of CTAB powder to the PAN solution and stirred for 12 h to form electrospinning solutions with increased conductivity [33]. Thus, two batches of solutions were prepared, PAN and PAN–CTAB. Subsequently, the solutions were poured into a 23-gauge syringe connected to a metal nozzle. The e-MB and discharged MB materials were used as substrates by sticking them on a drum collector. The e-MB material was immersed in IPA for 20 min, and then dried at room temperature for 24 h to nullify its electrostatic force. The electrospinning process conditions were as follows: the distance between the spinneret and the nonwoven substrates was 15 cm, the applied voltage from the power supply was 15 and 40 kV, the feed rate of the spinning solution was 5 μL/min, and the relative humidity (RH) and temperature were 40% ± 5%, and 22 ± 2 °C, respectively. Finally, the nanofiber web was dried in an oven at 100 °C for 2 h to evaporate the residual solvent.

### 2.3. Characterization of Nanofiber/Nanonet Web

The morphological characteristics of the PAN nanofiber web and PAN–CTAB nanofiber/nanonet web obtained with different voltages and substrates were investigated by field-emission scanning electron microscopy (FE-SEM, SU8010, Hitachi Co., Tokyo, Japan) at an acceleration voltage of 10 kV. The thermal properties of the two webs were analyzed by thermogravimetry (TGA, Q500, TA Instruments Co., New Castle, DE, USA) in an N_2_ atmosphere with a heating rate of 10 °C/min. The thermal behaviors were examined by differential scanning calorimetry (DSC, 404 C, Netzsch Co., Selb, Germany) from 30 to 350 °C with a heating rate of 10 °C/min under an N_2_ atmosphere. The crystal formation and structural characteristics due to the addition of CTAB were analyzed by X-ray diffraction (XRD, X’PERT-PRO Powder, PANalytical Co., Almelo, The Netherlands). The X-rays were generated at 40 kV and 30 mA with Cu–Ka radiation (wavelength, *λ* = 0.154 nm). The scan speed and measuring range were 4°/min and 10°–60°, respectively. The structural characteristics of the two types of webs were analyzed by Fourier transform infrared spectroscopy (FT-IR spectrometer, Nicolet NEXUS, Thermo Fisher Scientific Co., Waltham, MA, USA) using the ATR mode in the scan range of 400–4000 cm^−1^. The mechanical properties with and without CTAB were determined using a universal testing machine (Instron3343, Instron Co., Barcelona, Spain) equipped with a 50 N load cell. At least 10 rectangular web specimens (50 mm × 100 mm) were stretched at a constant crosshead speed of 10 mm/min and tested.

### 2.4. Analysis of Filter Media

The pore-size distributions and mean pore diameters of the PAN and PAN–CTAB filter media samples were measured by capillary flow porometry (CFP, CFO-1500AEX, Porous Materials Inc., Ithaca, NY, USA). All the samples were cut into 3 cm × 3 cm squares. Galwick with a surface tension of 20.1 dynes/cm was used as the wetting agent. Maximum volumetric flow rates of 20,000 and 100,000 L/m were used in the air permeability and pore distribution tests. The filtration performance of the media with the MB nonwoven material was tested using an automated filter tester (TSI 8130, TSI Instruments Co. Ltd., Shoreview, MN, USA). NaCl aerosols with a mass median diameter of 0.26 mm were produced using an atomizing air pump. The aerosol particles permeated the sample at a flow rate of 32 L/min. The filter media had an effective test area of 100 cm^2^. A mask was fabricated by adding spunbond nonwoven fabrics, which did not affect the filtering efficiency of the samples electrospun on the MB nonwoven fabrics. The size of mask sample was a width of 18 cm and a height of 17 cm, and the result was obtained by folding the middle part thrice. The filtering performance was tested with different air volumetric flow rates (10–80 L/min). Each sample was tested five times to ensure accuracy.

## 3. Results and Discussion

**Figure 2:**
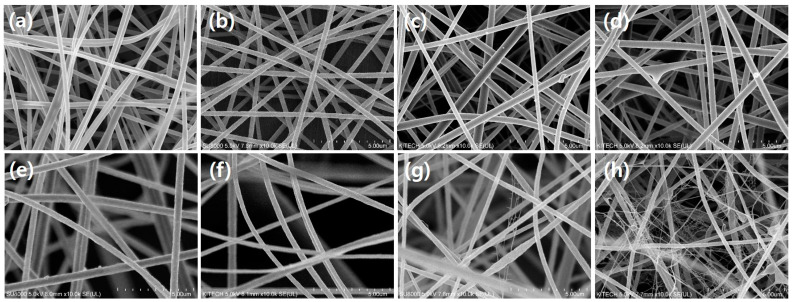
Field-emission scanning electron microscopy (FE-SEM) images of PAN (**a**–**d**) and PAN–CTAB (**e**–**h**) nanofiber/(nanonet) web with varying spinning voltage and substrate type: (**a**,**e**) 15 kV, meltblown (MB), (**b**,**f**) 15 kV, e-MB, (**c**,**g**) 40 kV, MB, and (**d**,**g**) 40 kV, e-MB.

A nanofiber filter media with nanonet structures was manufactured to improve its ability to filter ultrafine particles. To manufacture the PAN nanofiber web with nanonet structures, the effects of an ionic surfactant and spinning conditions were confirmed. Figure 2 illustrates the FE-SEM images of the PAN nanofiber web surface prepared according to the surfactant, substrate conditions (charged and uncharged MB substrates), and spinning voltages (15 and 40 kV). Figure 2a–d shows the surface images of the pristine PAN nanofiber web according to the manufacturing conditions. The formation of a nanonet under the applied voltage and substrate conditions was not observed. Figure 2e–f depicts the surface images of the PAN–CTAB nanofiber web or nanofiber/nanonet web prepared by adding CTAB as the surfactant. A nanonet was observed at an applied voltage ≥40 kV. The PAN–CTAB nanofiber/nanonet web spun on the e-MB substrate (Figure 2h) had a larger nanonet than that of the web spun on the discharged MB substrate. This result led to the conclusion that the ions in the e-MB substrate influence the nanonet production under high-voltage conditions (40 kV). The pristine PAN nanofiber web had an average diameter of 245.3 nm.

The average diameter of the PAN–CTAB nanofiber/nanonet web (Figure 2h) was 276.1 nm/26.1 nm. The addition of CTAB increased the diameter of the PAN nanofibers compared to that in the PAN nanofiber web.

**Figure 3:**
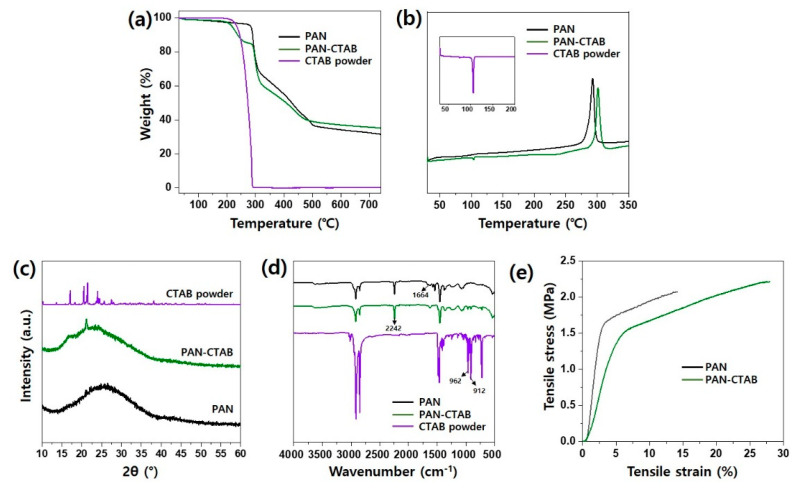
Characterizations of PAN nanofiber web, PAN–CTAB nanofiber/nanonet web, and CTAB powder. (**a**) Thermogravimetric analysis (TGA), (**b**) differential scanning calorimetry (DSC), (**c**) X-ray diffraction (XRD) patterns, and (**d**) Fourier transform infrared (FT-IR) spectra results of the three groups, respectively. (**e**) S–S curves of the PAN nanofiber and PAN–CTAB nanofiber/nanonet web.

TGA and DSC were performed to evaluate the effect of the thermal properties of CTAB on the PAN–CTAB nanofiber/nanonet web. The results of the TGA are illustrated in Figure 3a. The two types of nanofibers and CTAB powder obtained under each of the conditions began to decompose at 300.9, 229.18, and 284.81 °C. It was confirmed that the addition of CTAB lowered the temperature of thermal decomposition of the PAN–CTAB nanofiber/nanonet web. The DSC analysis confirmed the effect of CTAB powder on the thermal properties of the PAN nanofiber web. In general, the PAN nanofiber web generates exothermic peaks owing to cyclization, dehydration, and oxidation reactions at 200–350 °C [33]. During the DSC analysis, the C≡N bonds were converted to C=N bonds with the stabilization process [34]. The exothermic peaks of the PAN nanofiber web and PAN–CTAB nanofiber/nanonet web were confirmed at 295 and 300.2 °C, respectively (Figure 3b). The endothermic peak of the CTAB powder appeared at approximately 102 °C, and the presence of CTAB was confirmed by the occurrence of an endothermic peak at the same temperature in the PAN–CTAB nanofiber/nanonet web. The onset temperature of the PAN nanofiber web increased by approximately 5 °C with the addition of CTAB. CTAB acted as an inhibitor to retard the cyclization reaction of PAN in the PAN–CTAB nanofiber/nanonet web, and blocked the recombination reaction by reducing the free-radical formation rate of the nitrile group [34].

Figure 3c shows the XRD patterns of the pristine PAN nanofiber web, PAN–CTAB nanofiber/nanonet web, and CTAB powder. The PAN nanofiber web displayed a broad crystal peak at 25.7°. The crystal peak of the CTAB powder exhibited high intensities at 17.3, 20.4, 21.4, and 24.3°. The PAN–CTAB nanofiber/nanonet web showed a broad peak at approximately 17–25° under the influence of CTAB. It is plausible that the addition of the CTAB powder enlarged the PAN nanofiber web crystals.

Figure 3d displays the FT-IR spectrum of the pristine PAN nanofiber web, CTAB powder, and PAN/CTAB nanofiber/nanonet web. The spectrum corresponding to the pristine CTAB exhibits the characteristic absorption bands of the CTAB functional groups, as reported previously [6]. The absorption bands between 2960 and 2820 and 1510 and 1440 cm^−1^ correspond to –CH_2_– stretching and –CH_2_– bending in CATB and the PAN–CTAB nanofiber/nanonet web, respectively, confirming the presence of long alkyl groups of CTAB in the PAN–CTAB nanofiber/nanonet web [35]. The pristine PAN nanofiber web exhibited prominent peaks at 1452, 1664, 2240, and 2923 cm^−1^. The peaks at 1664, 2240, and 2923 cm^−1^ represent the stretching vibrations of –C=N, –C≡N, and –CH groups, respectively, whereas that at 1452 cm^−1^ represents the bending vibration of the –CH_2_ group. A subtle increase in the characteristic peak of the PAN nanofiber web was observed at 1664 cm^−1^ with the addition of CTAB in the PAN nanofiber web.

Figure 3e summarizes the mechanical properties of the pristine PAN nanofiber web and PAN–CTAB nanofiber/nanonet web. The tensile strengths of the two webs were approximately 2.07 and 2.27 MPa, respectively. The tensile strength of the PAN–CTAB nanofiber/nanonet web improved by approximately 110% with the addition of the CTAB powder over that of the pristine web. The elongation of the PAN–CTAB nanofiber nanonet web also improved by approximately 200% compared with that of the pristine PAN nanofiber web. Notably, the tensile strain nearly doubled because the nanonet was distributed between nanofibers and served as a reinforcement in the cross-sectional direction [2].

**Figure 4:**
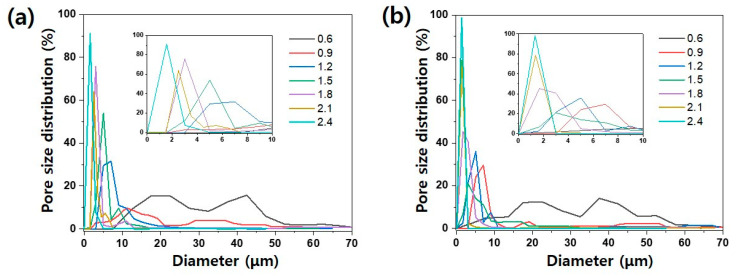
Pore-size distribution of (**a**) PAN and (**b**) PAN–CTAB filter media with varying base weight of the electrospun web.

Figure 4a,b shows the pore-size distribution of the filter media fabricated by laminating the PAN nanofiber web and PAN–CTAB nanofiber/nanonet web by weight (0.6–2.4 g/m^2^) on the MB substrate. As the weight of the nanofiber web increases, leading to thickening of the nanofibers, the average pore size decreases as the pores are gradually blocked. In addition, in the case of the PAN–CTAB filter media with the net structure, the pore-size distribution was confirmed to be narrower than that of the PAN filter media owing to nanonet generation. The inset graph, an enlargement of the region corresponding to <10 μm diameter in the original graph, shows that the smallest pore size is attained at 0.9 g/m^2^ in the case of the PAN–CTAB nanofiber/nanonet web.

**Figure 5:**
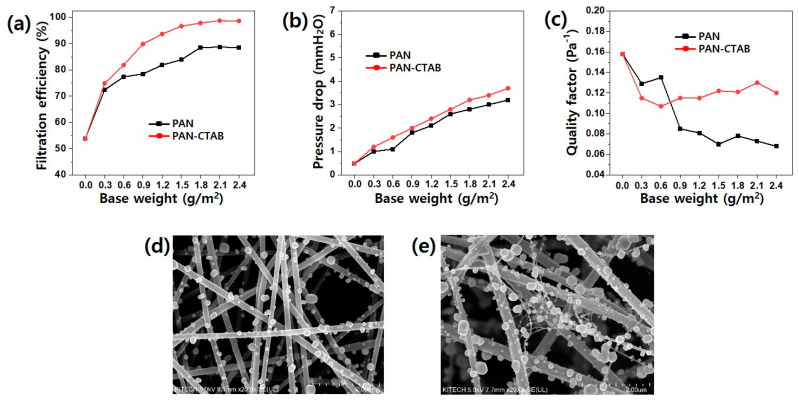
(**a**) Filtration efficiency, (**b**) pressure drop, and (**c**) quality factor of the PAN and PAN–CTAB filter media for different base weights of the electrospun web. The SEM images of (**d**) PAN nanofiber and (**e**) PAN–CTAB filter media after five iterations of the TSI8130 filtration evaluation test.

Despite its high efficiency, the application of a nanofiber filter as a mask is limited by its high breathing resistance. The filtration efficiency was evaluated because it was affected by the formation of the nanonet structure in the nanofiber. Figure 5a shows the filtering efficiency according to the nanofiber weight. The control MB group without nanofibers had a filtering efficiency of 53.8%, and the filter media laminated with the PAN nanofiber web had an increased filtration efficiency of 72.4–88.7% as the weight of the nanofiber increased to 0.3–2.4 g/m^2^. The efficiency of the filter media with stacked PAN–CTAB nanofiber/nanonet webs also improved to 74.9–99%. As the filter efficiency increased, the pressure drop also increased to 0.489 (only MB) up to 3.2 mmH_2_O for the media laminated with the PAN nanofiber web, and to 0.489 (only MB) up to 3.7 mmH_2_O for the media laminated with the PAN–CTAB nanofiber/nanonet web. Based on these results, the quality factor (QF = −ln(1 − η)/Δp, where η is the filtration efficiency, and Δp is the pressure drop) was calculated to evaluate the comprehensive filtering performance [36].

The QF is an indicator of the filtering efficiency considered for the pressure drop in the filter media. The filter in which the PAN–CTAB nanofiber/nanonet web was stacked from 0.9 g/m^2^ or higher by weight of the laminated nanofiber had a significantly higher QF. The SEM images in Figure 5d,e confirm NaCl aerosol capture on the nanofiber after the filtration test. A notably larger amount of NaCl was adsorbed to the filter media in which the PAN–CTAB nanofiber/nanonet web was laminated than that on which the pristine PAN nanofiber web was laminated. This is because its ability to trap particles was improved by the presence of the nanonet between large pores.

**Figure 6:**
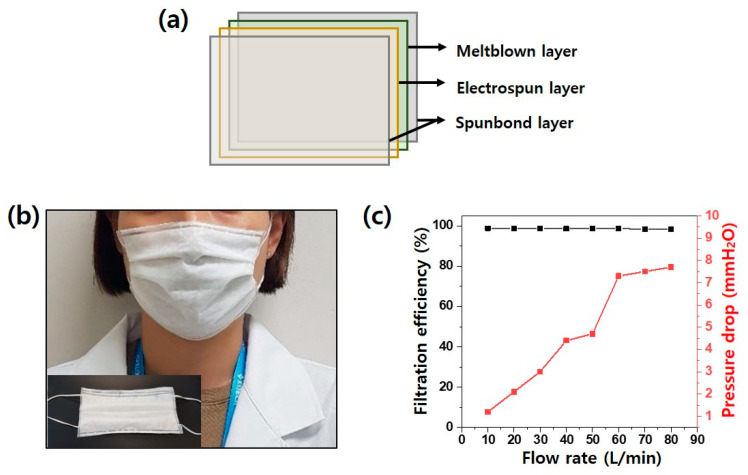
Schematic diagram of the structure of the filter media (**a**). A photograph of a person wearing the fabricated mask (**b**). Filtration performance evaluation by flow rate (**c**).

A mask prototype was fabricated with a PAN–CTAB nanofiber/nanonet web-layered sample of 2.1 g/m^2^, which had the highest QF value (0.13 Pa^−1^) among the filter media. As shown in the schematic diagram of Figure 6a, the mask was prepared by adding spunbond nonwoven fabric on both sides of the sample, which did not affect the filtering efficiency (Figure 6b). The filtering efficiency was tested with various flow rates. Consequently, the pressure drop across the layers of the mask gradually increased at high flow rates, and the efficiency was maintained at 98.5–99%. When the flow rate reached 80 L/min, the pressure drop was only 7.7 mmH_2_O, which indicates high efficiency.

## 4. Conclusions

PAN–CTAB filter media containing a nanofiber/nanonet web were prepared using a cationic surfactant to increase the ion mobility via electrospinning at a high voltage. A larger nanonet was formed on an MB nonwoven substrate when the applied electrospinning voltage was 40 kV. The residual amount at 750 °C was found to be 4.5% more than that of the PAN–CTAB nanofiber/nanonet web. The DSC results showed that the addition of CTAB improved the thermal properties of the web as the exothermic peak temperatures increased by 5 °C. In the sample with the nanonet formed by the addition of CTAB, application of high voltage (40 kV), and use of the e-MB substrate, the nanofiber diameter increased; in addition, the tensile strength and tensile strain increased by 110% and 200%, respectively. The filtration efficiencies of the PAN (nanofiber web laminated on MB) and PAN–CTAB filter media were 88.7% and 99%, respectively, at the largest weight (nanofiber/nanonet web). The sample with the highest QF (0.13 Pa^−1^) among the filter media was a 2.1 g/m^2^ stack of PAN–CTAB nanofiber/nanonet web. A high efficiency, low-pressure-drop (3.4 mmH_2_O) filter, with a filtration efficiency of 99%, was achieved. For application in a face mask, the uniformity of human inhalation and exhalation was considered, and a high filtration efficiency was measured at a high flow rate (80 L/min). The PAN–CTAB filter media have markedly improved properties compared to those of the PAN filter media of the control groups. In summary, the specifications of the PAN nanofiber/nanonet web can be altered by controlling the process parameters.

## Data Availability

Not applicable.
